# Anisotropic scaling for 3D topological models

**DOI:** 10.1038/s41598-021-01888-x

**Published:** 2021-11-18

**Authors:** S. Rufo, M. A. R. Griffith, Nei Lopes, Mucio A. Continentino

**Affiliations:** 1 Beijing Computational Science Research Center, Beijing, 100193 China; 2 CeFEMA, Instituto Superior Técnico, Universidade de Lisboa, Av. Rovisco Pais, 1049-001 Lisbon, Portugal; 3 Centro Brasileiro de Pesquisas Físicas, Rua Dr. Xavier Sigaud, 150 - Urca, Rio de Janeiro, RJ 22290-180 Brazil; 4 Departamento de Física Teórica, Universidade do Estado do Rio de Janeiro, Rua São Francisco Xavier 524, Maracanã, Rio de Janeiro, RJ 20550-013 Brazil

**Keywords:** Phase transitions and critical phenomena, Topological matter

## Abstract

A proposal to study topological models beyond the standard topological classification and that exhibit breakdown of Lorentz invariance is presented. The focus of the investigation relies on their anisotropic quantum critical behavior. We study anisotropic effects on three-dimensional (3D) topological models, computing their anisotropic correlation length critical exponent $\nu$ obtained from numerical calculations of the penetration length of the zero-energy surface states as a function of the distance to the topological quantum critical point. A generalized Weyl semimetal model with broken time-reversal symmetry is introduced and studied using a modified Dirac equation. An approach to characterize topological surface states in topological insulators when applied to Fermi arcs allows to capture the anisotropic critical exponent $\theta =\nu _{x}/\nu _{z}$. We also consider the Hopf insulator model, for which the study of the topological surface states yields unusual values for $\nu$ and for the dynamic critical exponent *z*. From an analysis of the energy dispersions, we propose a scaling relation $\nu _{\bar{\alpha }}z_{\bar{\alpha }}=2q$ and $\theta =\nu _{x}/\nu _{z}=z_{z}/z_{x}$ for $\nu$ and *z* that only depends on the Hopf insulator Hamiltonian parameters *p* and *q* and the axis direction $\bar{\alpha }$. An anisotropic quantum hyperscaling relation is also obtained.

## Introduction

Landau’s theory of phase transitions^1^ provides the standard approach to describe transitions between different states of matter in condensed matter and statistical physics. However, it is not suitable to describe systems that separate phases of matter containing different electronic Bloch states topology^2–6^, also called topological phase transitions (TPTs). In this case there is no symmetry breaking associated with an ordered phase when the system undergoes a TPT^6^. For this reason, many different approaches have been proposed recently to identify the universality classes of topological transitions using scaling ideas^6–18^.

In addition, in the late 1990s, it was established the classification table.

1 warning for topological insulators (TIs) and superconductors (SCs)^19,20^ that contributed to make this topic a fruitful and exciting area of research. Nevertheless, it is worth to emphasize that there are still many open questions, such as, the fact that some topological phases are beyond this standard classification. For instance, the gapless topological phase and the intrinsic topological (IT) phases^21^. The latter only preserves U(1) charge conservation symmetry and possesses translational symmetry that makes momentum-k a good quantum number^22,23^. IT materials also can exhibit exotic excitations what makes them a very interesting and attractive branch of research^22–28^.

This research topic is closely related to applications since the discovery of new materials can lead to the development of new devices. Nowadays, there are many technological proposals based on topological materials^29–31^. The most prominent applications for TIs rely on their properties of carrying an electric current only at the edges or surfaces in two (2D) and three-dimensional (3D) case respectively, while the bulk remains an insulator^2,4^.

Beyond the standard topological materials, we point out the Dirac semimetals (DSMs) that present band touching at some points in their band structures. These are protected by time-reversal symmetry (TRS) and/or inversions symmetry (IS)^32^. For this reason, DSMs are recognized to be an intermediate phase between a usual insulator and a TI phase. After a breaking of TRS the Dirac nodes are divided into two nodes, which are called Weyl nodes^33^. In a Weyl semimetal crystal, the chiralities associated with the Weyl nodes (Fermi points) can be understood as topological charges. These leads to monopoles and anti-monopoles of Berry curvature in momentum space, which serve as the topological invariant of this phase^34^. Another property, induced by the projection of the bulk Weyl nodes is the appearance of *Fermi arcs* in the surface of the Brillouin zone^34,35^. A non-trivial value of the sectorial Chern number ${\mathscr {C}}(k_{z})$ guarantees that there are chiral surface states. These form Fermi arcs that connect the projections of two Weyl points with opposite topological charges onto the surface of the Brillouin zone^34^.

Although Dirac semimetals also exhibits Fermi arcs, in the Weyl case these are bulk related while in the Dirac case this is not always true^36^. The Weyl nodes with zero energy observed in Weyl semimetal materials (WSMs) can be described as low-energy fermion excitations using the Dirac equation^37–39^.

The band touching in topological semimetals usually exhibits a linear behavior. Nevertheless, Ref.^40^ shows that $\hbox {SrSi}_2$ is a WSM that exhibits a quadratic dispersion. This result inspires us to investigate non-linear band touching, i.e., that exhibits a breakdown of Lorentz invariance^6,7^ and consequently is in a different universality class. In this case, the notion of anisotropic scaling becomes fundamental. This is characterized by the ratio $\theta =\nu _{//}/\nu _{\bot }$, where $\nu _{//}$ and $\nu _{\bot }$ denote the correlation length critical exponents along different directions^2,41^.

There is another class of topological materials that is not protected by any discrete symmetry (time reversal, chiral and particle-hole symmetries), represented by IT materials. A special case arises for two band systems, which may realize a *Hopf insulator phase*. Due to this non-symmetry protected character, a non-trivial Hopf mapping, for *m* filled and *n* empty bands $m=n=1$, makes the Grassmannian manifold $G_{m,m+n}({\mathbb {C}})=G_{1,2}({\mathbb {C}})$ topologically equivalent to ${\mathbb {S}}^2$^22,42,43^. From this mapping procedure arises two parameters *p* and *q* for which there is a corresponding tight-binding Hamiltonian^22^. If *p* and *q* are integers prime to each other (greatest common divisor equal to one) this Hamiltonian describing the Hopf insulator can be explicitly obtained^22,44^.

In this work, we identify different universality classes for anisotropic 3D topological models beyond the standard topological classification, such as, the Weyl semimetal^39^ and the Hopf insulator model^22^. We also introduce an generalized Weyl semimetal model to investigate the correlation length exponent $\nu$ along any direction. Considering these models, we perform a study of topological surface states to determine the correlation length critical exponent $\nu$, and the dynamic critical exponent *z*. The former is obtained directly from numerical calculations of the penetration length of the surface states. This length is the characteristic length of the topological phase transition and can be identified as the correlation length. It depends on the distance to the transition and diverges as $\xi \propto |M|^{-\nu }$ where *M* is the distance to the topological critical point^6,17^.

We use a modified Dirac equation^45^, containing quadratic corrections in *momentum*, to show that the study of topological surface states is adequate to describe anisotropic features, even for 3D topological models. We show that the only requirement, once there is a non-trivial wave-function solution, is that the surface states only decay into the bulk.

In special, for the Hopf insulator model we investigate the energy dispersion relations and propose a scaling law $\nu _{\bar{\alpha }}z_{\bar{\alpha }}=2q$, where the critical exponents $\nu$ and *z* depend on only on the parameters *p* and *q* and the axis ($\bar{\alpha }=x,\,y,\,z$) that holds the decaying surface state. We also obtain the *anisotropic critical scaling exponent* in the form $\theta =\nu _{x}/\nu _{z}=z_{z}/z_{x}$. We observe that very exotic critical exponents may be possible depending on each pair [*p*, *q*]. This is confirmed numerically from the decay of the topological surface state, that for the pair $[p,q]=[1,3]$ yields $\nu _{x}=3$ and $z_{x}=2$.

In general, our results exhibit a connection between the properties of zero-energy surface states and the critical phenomena in the bulk for TPTs beyond the standard classification.

## Weyl semimetal model

The Weyl semimetal model with broken time-reversal symmetry ${\mathscr {T}}$ is well-known^34,37,46^. In a cubic lattice, it can be described by the Dirac equation as follows,

1 $$\begin{aligned} {\mathscr {H}}(k) = t_{x}\sin {k_{x}}\tau _{x}+t_{y}\sin {k_{y}}\tau _{y} + t_{z}(\gamma +2-\cos {k_{x}}-\cos {k_{y}}-\cos {k_{z}})\tau _{z} \end{aligned}$$

where $t_i$ are the hopping terms, $\tau _{i}$ are the Pauli matrices in orbital space and $\gamma$ is the control parameter of the TPT. Eq. (1) describes two Weyl nodes at positions $\mathbf {k}_{0}=\{0,0,\pm k_{0}\}$ where $k_0=\cos ^{-1}\gamma$ with $-1<\gamma <1$. When $\gamma =1$, $k_0=0$ and the two Weyl points merge at a topological phase transition.

Expanding Eq. (1) around the Weyl point $\mathbf {k}_{0}$, following Eq. (17) (refer to ’Methods’ section for details) we obtain the energy projections in $k_{y}$ and $k_{z}$ directions.

For the projection in $k_{y}$ direction, we take $k_{x}=0$, and at the phase transition point $\gamma =1$ we have

2 $$\begin{aligned} E(k_{y})=\pm \sqrt{t_{y}^{2}k_{y}^{2}+t_{z}^{2}\frac{k_{y}^{4}}{4}}. \end{aligned}$$

This implies a linear crossing behavior of Eq. (2), that is, $E(k_{y})\propto t_{y}k_{y}$, as we approach the phase transition, since the quartic term $k_{y}^{4}$ goes to zero more quickly than the quadratic one $k_{y}^{2}$. The form of dispersion at the transition defines the dynamic exponent $z_y$ by $E \propto k^{z_y}$ and we can identify the dynamic exponent $z_y=1$.

On the other hand, in the $k_{z}$ direction, for $k_{y}=0$, the energy projection becomes

3 $$\begin{aligned} E(k_{z})=\pm t_{z} \left[ \sin {k_{0}}(k_{z}-k_{0})-\frac{\cos {k_{0}}}{2}(k_{z}-k_{0})^{2}\right] . \end{aligned}$$

Note that, we can rewrite the energy dispersion in Eq. (3) as $E(k_{z})=A_{1}(k_{z}-k_{0})+A_{2}(k_{z}-k_{0})^{2}$ with $A_1=t_z \sin k_0$ and $A_2 =-t_z\frac{\cos k_0}{2}$. Accordingly, in a way similar to what happens in the extended SSH model in Ref.^6^, close to the critical point $\gamma =1$ the coefficients are $A_{1}=0$ while $A_{2}\ne 0$. This means that the quadratic term dominates and ensures a quadratic behavior $E(k_{z})\propto \frac{t_{z}}{2}(k_{z}-k_{0})^{2}$ in the vicinity of the phase transition. This relation also allows to identify the dynamic critical exponent $z_z=2$.

**Figure 1:**
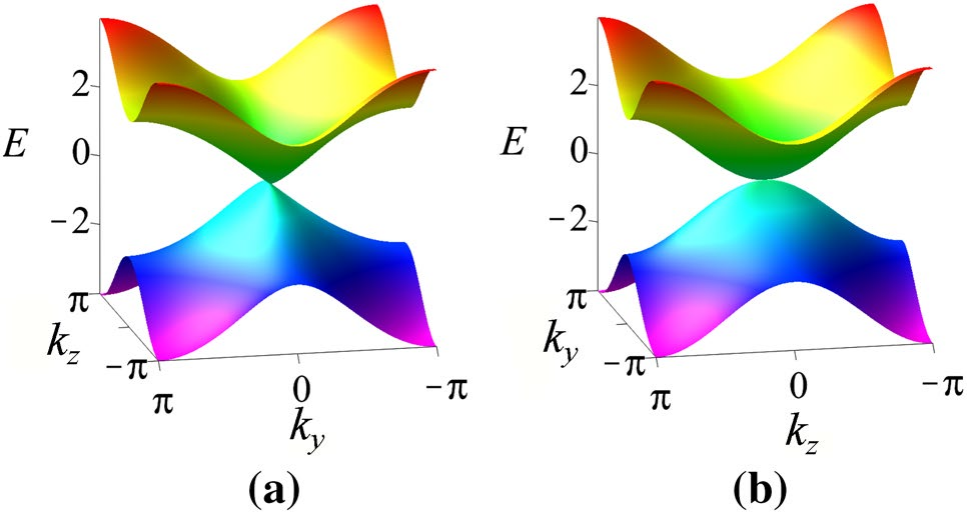
Anisotropic bands dispersion at the critical point $\gamma =1$ for $k_{x}=0$. (**a**) Along $k_{y}$ and (**b**) along $k_{z}$ with linear and quadratic band-crossing, respectively^47,48^.

In Fig. 1 we call attention to this anisotropic band-crossing behavior, linear along the $k_{y}$ (Fig. 1a) and quadratic along $k_{z}$ direction (Fig. 1b). It is well known that this anisotropic way of behaving has impact over the critical exponents along each direction. For this reason, we study the penetration depth of the surface states of the Weyl semimetal model in order to obtain the critical exponents $\nu$ and *z*.

### Topological surface states study

**Figure 2:**
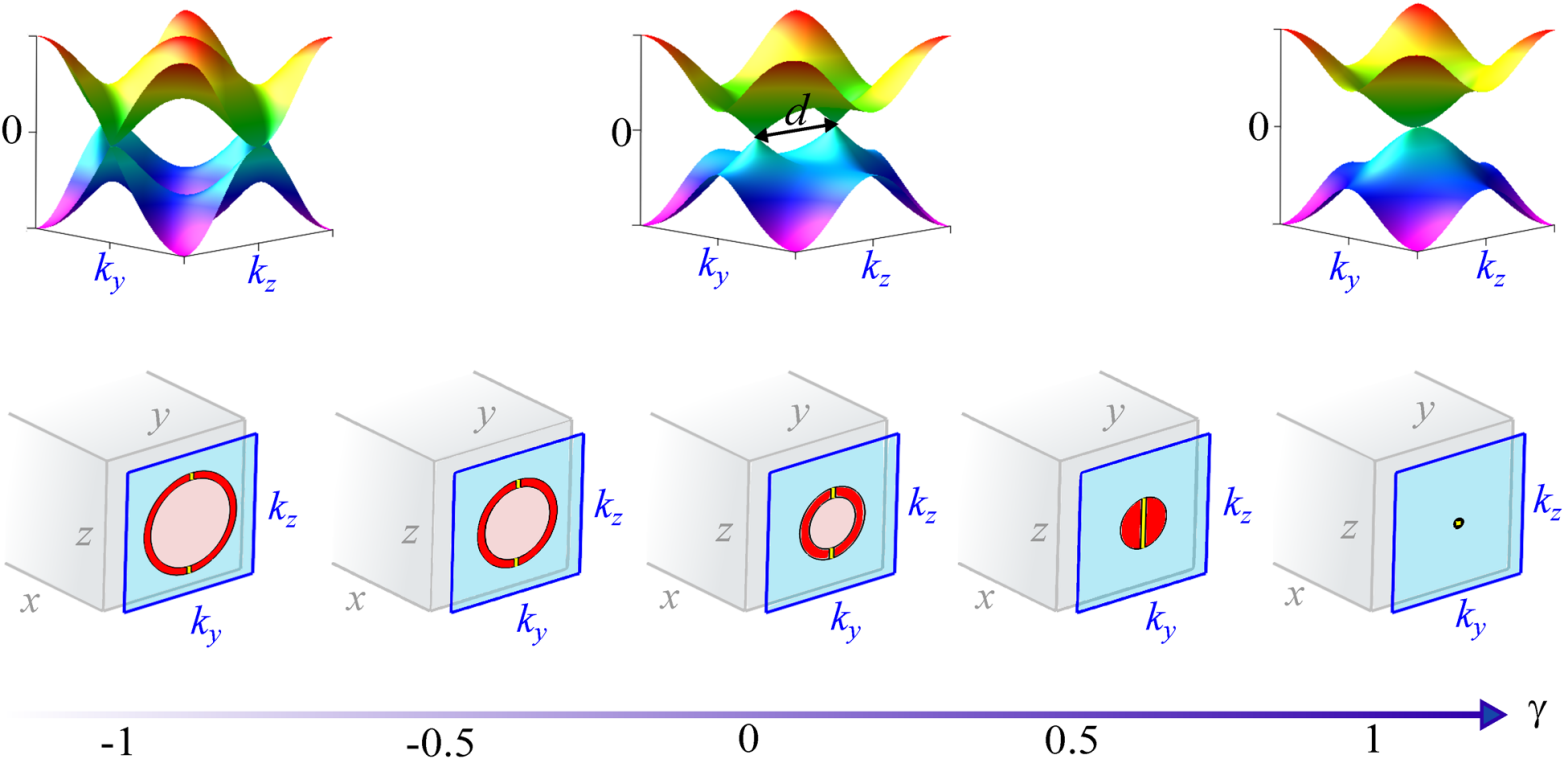
Weyl points and surface states with energy $\varepsilon =\pm t_{y}k_{y}$ from $\gamma =-1$ (creation of Weyl points) to $\gamma =1$ (annihilation of Weyl points) (bottom blue arrow). (top figure) Bulk band energy for $\gamma =-1,\,0,\,1$, where *d* is the distance between the Weyl points. (bottom figure) The gray shape represents the material in real space coordinates *x*, *y*, *z*. Accordingly, there are two momenta space planes ($k_{y}-k_{z}$) ($k_{x}-k_{z}$) for *x* (*y*) open-boundary conditions, where each plane holds the surface state related to the edge of the material along this open direction. In perspective, to exemplify the *x* open-boundary conditions case, we depicted the momentum space $k_{y}-k_{z}$. In this momentum space, we have solutions where the surface states only decay with $\lambda _{-}=a$ real and positive (red region) which is satisfied for $1-2\gamma< k_{y}^{2}+k_{z}^{2}<2(1-\gamma )$ Eq. (8), solutions where the surface states decay and oscillate with $\lambda _{-}=a\pm ib$ for $a>0$ (pink region), as well as, solutions where the surface states only oscillate (blue region). We also have decaying zero-energy surface states along $k_{y}=0$ (yellow stripes). In special, at $\gamma =0.5$ the pink color region vanishes and we observe surface states that only decay. We note, as $\gamma \longrightarrow 1$ that the radius of the circle containing the surface states decreases until becomes a point at the critical point $\gamma =1$^47,48^.

To obtain the topological surface states behavior, we need to consider the presence of quadratic terms in *k* in Eqs. (2) and (3) that make explicit the anisotropic behavior of the band-crossing. Then, to deal with Eq. (1) it is useful to consider the modified Dirac equation in 3D^45^

4 $$\begin{aligned} H(\mathbf {p})=vp_{x}\alpha _{x}+vp_{y}\alpha _{y}+vp_{z}\alpha _{z}+(M v^2-Bp^2)\beta \end{aligned}$$

where, $\mathbf {p}=\hbar \mathbf {k}$ ($\hbar \equiv 1$), $v=1$, with $\alpha _{i}$ and $\beta$ are the Dirac matrices, *M* is the rest mass, *B* carries the quadratic correction in *k* and $p^{2}=p_{x}^{2}+p_{y}^{2}+p_{z}^{2}$. In addition, the elements *M* and *B* allow to distinguish between the trivial ($M B<0$) and non-trivial ($M B>0$) topological states^45^.

Next, we put Eq. (1) in the same form of Eq. (4), in terms of $\gamma$ and expanding directly around the critical wavevector $\mathbf {k}_{0}=(0,0,0)$, we have

5 $$\begin{aligned} H(\mathbf {k})= t_{x}k_{x}\tau _{x}+t_{y}k_{y}\tau _{y}+\left[ M-B\left( k_{x}^{2}+k_{y}^{2}+k_{z}^{2}\right) \right] \tau _{z}, \end{aligned}$$

where $M=t_{z}(\gamma -1)$, $B=-\frac{t_{z}}{2}$. From now on, $t_{\bar{\alpha }}>0$ ($\bar{\alpha }=x,y,z$) and for this reason B is negative. This means that we have trivial and non-trivial topological phases for $M>0$ and $M<0$, respectively. In fact, the non-trivial $M<0$ phase region shelters the two Weyl nodes. The point $M=0$, or $\gamma =1$ is the critical point at which the topological transition occurs. The Dirac matrices were replaced by the Pauli matrices in Eq. 5 because the problem has just orbital degrees of freedom and we have two decoupled equations^49,50^.

The investigation of the topological surface states along each direction, requires choosing one direction at a time and considering open-boundary conditions for it. It is important to note that the anisotropy among the momentum directions provides different wave-function behavior as well as different penetration lengths of the surface states in each case.

### Surface state conditions

We obtained similar behavior for the wave-functions and penetration depths for open-boundary conditions along *x* and *y* directions. This is expected since in Eq. (5) these directions are equivalent. On the other hand, no non-trivial wave-function for the surface state is obtained when *z* is under open-boundary conditions, see Eq. (34) (refer to ’Methods’ section for details). It is reasonable, since this last direction shelters the Weyl points and any projection on a perpendicular plane returns a point.

So, here we consider open-boundary conditions along the *x* direction (refer to ’Method’ section for details). In Eq. (30), we present the proper wave-function and its characteristic decay length $\xi _{\pm }=\lambda _{\pm }^{-1}$, see Eq. (26). In order to specify which quantity $\xi _{\pm }$ truly represents the penetration length of the surface state, we study the limiting behavior of these lengths in the vicinity of the critical point. From Eq. (26), for $\gamma \longrightarrow 1$ and $k^{2}\longrightarrow 0$ we obtain $\lambda _{+}\rightarrow \frac{t_{x}}{B}$ and $\lambda _{-}\rightarrow 0$. Since the correlation length must diverge at the phase transition, the length to be identified as the penetration depth or correlation length is $\xi _{-}=\lambda _{-}^{-1}\longrightarrow \infty$.

If the surface state only decays in the bulk, the penetration depth $\xi _{-}$ must be a purely real and positive quantity. This means that Eq. (26) must satisfy

6 $$\begin{aligned} \frac{t_{x}}{2|B|}\left[ 1 - \sqrt{1-\frac{4MB}{t_{x}^{2}}+ \frac{4B^{2}{\bar{k}}^{2}}{t_{x}^{2}}}\right]> & {} 0 \end{aligned}$$

7 $$\begin{aligned} t_{x}^{2}-4MB+4B^{2}{\bar{k}}^{2}> & {} 0 \end{aligned}$$

that returns a narrow interval for ${\bar{k}}^2=k_{y}^{2}+k_{z}^{2}$ given by

8 $$\begin{aligned} 1-2\gamma< k_{y}^{2}+k_{z}^{2}<2(1-\gamma ). \end{aligned}$$

Beyond that, the surface states are propagating excitations with energy $\varepsilon =\pm t_{y}k_{y}$.

In Fig. 2, we show the Weyl points (top figure) and the surface states in momentum space $k_{y}-k_{z}$ for open-boundary conditions along the *x* direction (bottom figure). Each blue plane represents the perspective of momentum space with respect to real space, denoted by the gray shape. Along the blue plane, we present the evolution of the surface states in red, pink and yellow colors, as a function of $\gamma$. In red color, we have the region where each pair ($k_{y},k_{z}$) corresponds to a surface state that only decays into the bulk. For this, the condition of Eq. (8) must be satisfied, i.e., $\lambda _{-}=a$ is real and positive. In pink color, we have the situation where the surface states decay and oscillate with $\lambda _{-}=a\pm ib$, where $a>0$ and $b>0$ ensure the decay and oscillatory behavior, respectively. The yellow stripes also denote surface states which are purely decaying in the bulk, but distinctively from the region in red these are zero energy states. Accordingly to Fig. 2, as we approach the critical point $\gamma =1$, the region of surface states decreases until becomes a point. Along this process, we also identify a change of behavior at $\gamma =0.5$. As $\gamma$ increases beyond this point, the pink area is no longer observed and just the red region and the yellow stripes, both associated with surface states that only decay into the bulk, remain.

### Surface states and critical exponent $\nu$

Notice from Fig. 2 that the only point in the domain of surface states that persists from $\gamma =0.5$ to the critical point $\gamma =1$ is the pair $(k_{y},k_{z})=(0,0)$. So, this is the only point that allows to verify the behavior of the penetration depth in the vicinity of criticality.

**Figure 3:**
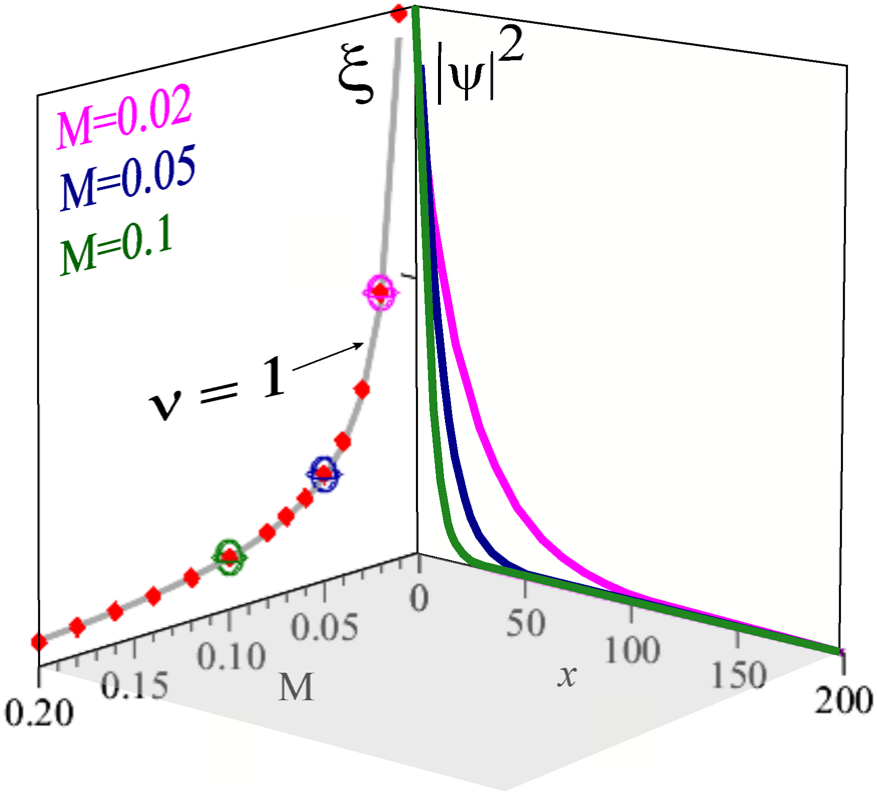
Decay of the probability density in the bulk and the penetration depth $\xi _{-}$ as function of the distance to the critical point. In the plane $x-|\psi |^2$, we present the decay behavior of the wave-function into the bulk along *x* direction, for the distances to the critical point $M=0.1,\,0.05,\,0.02$, in green, blue and magenta solid lines, respectively. From these plots we can extract $\xi _{-}$, using $|\psi |^2(x= \xi _{-})=e^{-1}$. In the plane $M-\xi$, we plot these values of the correlation length (red diamonds) as a function of the distance to the critical point *M* and note that this is well described by $\xi _{-} = M^{-\nu }$ with $\nu =1$ (gray solid line). For clarity in the plane $M-\xi$ we use the same color scheme of the plane $x-|\psi |^2$ to highlight the corresponding red diamond symbol^47,48^.

Accordingly, to perform a study of the topological surface state for this TPT we consider the probability density $|\psi |^2$ and obtain the site *n* in the *x* direction where this has decayed to $e^{-1}$ of its value at the surface ($n=1$)^6^. This allows to obtain the penetration length $\xi _{-}$ as a function of the distance to criticality and consequently to determine the critical exponent $\nu$ that controls its divergence.

Figure 3, shows the penetration depth $\xi _{-}$ as a function of the distance *M* to the critical point with open-boundary conditions along the *x* direction. In the plane $M-\xi$, the behavior of the penetration depth $\xi _{-}$ (red diamonds) as a function of *M* can be fitted perfectly well by the expression $\xi =|M|^{-\nu }$ with the correlation length exponent assuming the value $\nu =1$ (gray curve). This determines unambiguously that $\nu =1$ along the *x*-direction.

We plot the planes $\xi -M$ and $|\psi |^2-x$ in perspective to show clearly the connection between the divergence of the penetration depth (plane $M-\xi$) with the degree of delocalization of the surface state, (plane $x-|\psi |^2$). As we approach the critical point the surface state becomes less delocalized. Then we have identified a diverging length at the topological transition in the Weyl semi-metal, namely the penetration depth of the surface state in the non-trivial topological phase.

## Generalized Weyl model: emergence of Fermi arcs in all surfaces

In previous sections, we were able to obtain the critical exponents $\nu _{x}=\nu _{y}=1$ from the study of the penetration length of the topological surface states. The dynamic exponents, $z_{x}=z_{y}=1$ were, in turn, obtained from an analysis of the dispersion relations at the topological transition, Eq. (2). The numerical calculation of the correlation length exponents require the use of open-boundary conditions along the isotropic *x* and *y* directions. However, in the *z*-direction there is no non-trivial wave function solution of the Dirac equation. Although the analysis of the energy dispersion in the *z* direction, Eq. (3) yields a dynamic critical exponent $z_{z}=2$, we are not able to evaluate the correlation length exponent $\nu _{z}$ along this direction.

**Figure 4:**
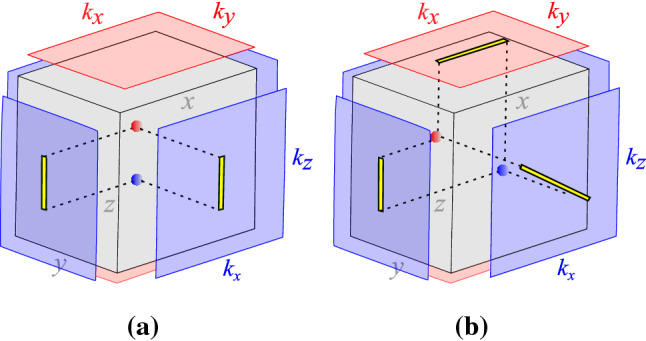
Schematic representation of the zero-energy Fermi arcs. (**a**) Fermi arcs for the Weyl semimetal model Eq. (1), with Weyl point projections only in the planes $k_{x}-k_{z}$ and $k_{y}-k_{z}$ (four light blue planes). (**b**) Fermi arcs for the generalized model Eq. (9), where the Weyl point projections are now present in all planes in momentum space, including the projection along the plane $k_{x}-k_{y}$ (two light red planes). Note that, the additional term $t_{a}\sin {k_{z}}$ promotes a rotation of the Weyl point projection around the $k_{y}$ direction. This allows the calculation of the penetration depth also in the *z* direction in case (**b**). For simplicity, we show only the projections along the three independent planes^47,48^.

In order to overpass this, based on the Weyl Hamiltonian of Eq. (1) we propose an generalized Hamiltonian given by,

9 $$\begin{aligned} {\mathscr {H}}(k) = {\mathscr {H}}_{Weyl}(k)+t_{a}\sin {k_{z}}\tau _{x}, \end{aligned}$$

where ${\mathscr {H}}_{Weyl}(k)$ recovers the Weyl Hamiltonian of Eq. (1). Since we have just added the term $t_{a}\sin {k_{z}}$ to the $\tau _{x}$ in the Weyl Hamiltonian, see Eq. (1), this addition does not break any additional symmetry (refer to ’Methods’ section for details). For this reason, Eq. (9) still describes two Weyl nodes but along a different plane, as we will discuss below. In special, we choose $t_{a}=\sqrt{\mid 1-\gamma \mid }$ that does not affect the anisotropic character of the energy spectrum and ensures the same critical parameter as before, $\gamma =1$. This additional term allows us to obtain a non-trivial wave-function in all three directions under open-boundary conditions, including the *z*-direction. As a consequence, we can obtain Fermi arcs along all surface planes in momentum space, see Fig. 4.

In Fig. 4, we compare the emergence of the Fermi arcs for the Weyl semimetal model, Eq. (1), in Fig. 4a with the Fermi arcs for the generalized Weyl model, Eq. (9), in Fig. 4b. We represent the Weyl points as the blue and red spheres to evidence the positive and negative topological charges, respectively. In Fig. 4a, we can observe the projections of the Weyl points only along the plane $k_{x}-k_{z}$ and $k_{y}-k_{z}$ (four light blue planes), since the Weyl points are along the *z* direction. On the other hand, in Fig. 4b, we also can see the projections along the plane $k_{x}-k_{y}$ (two light red planes), besides the other planes. This happens because the Weyl points now belong to the plane $x-z$ (for $y=0$ inside the cube), and they are not aligned along the *z* direction, as before. The presence of the term $t_{a}\sin {k_{z}}$ ensures the rotation of the Fermi arcs around the $k_{y}$ direction. We highlight in Fig. 2 the Fermi arcs as yellow stripes, which account for all zero energy, purely decaying surface states.

**Figure 5:**
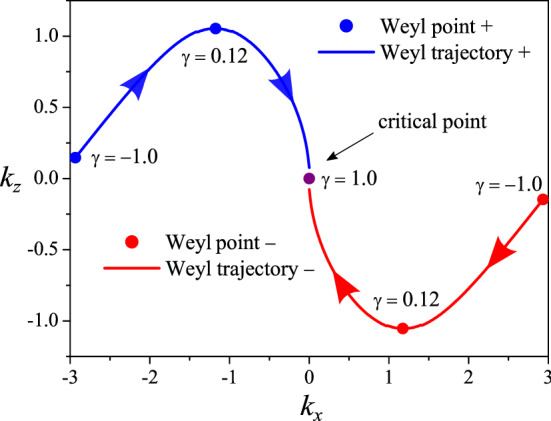
Trajectory of the Weyl points along the $k_{x}-k_{z}$ plane for *y* direction with open-boundary conditions. The blue and red circles denote the Weyl points with positive and negative topological charges, respectively, and the solid lines indicate the corresponding Weyl trajectories. The arrows indicate the paths of the Weyl points from $\gamma =-1.0$, going through $\gamma =0.12$, until the critical point at $\gamma =1.0$ where the Weyl points collide (purple circle)^47,48^.

Next, we perform an analysis of the topological surface states in the generalized Weyl model. This now yields decay exponents $\lambda _{\pm }$ for open-boundary conditions along any direction (refer to ’Methods’ section for details). The main change in $\lambda _{\pm }$ occurs for open-boundary conditions along *x* and *z* directions, where an additional term shows up inside the square root in Eqs. (38) and (39), respectively. In both cases the energy remains $\varepsilon =\pm sgn(B)t_{y}k_{y}$ and therefore the zero-energy surface states preserve the linear behavior along $k_{y}=0$, as obtained for the Weyl semimetal model. Otherwise, for *y* open-boundary conditions $\lambda _{\pm }$ is the same of Eq. (32) and the change occurs at the energy of the surface states now given by $\varepsilon =\pm sgn(B)(t_{x}k_{x}+t_{a}k_{z})$ (refer to ’Methods’ section for details). The zero-energy solutions correspond to the tilted Fermi arc along the plane $k_{x}-k_{z}$ in Fig. 4b with linear coefficient $-(t_{x}/t_{a})$. We also verify that these Fermi arcs correspond to the zero-energy surface states obtained numerically by diagonalization of the Hamiltonian of Eq. (9), for a lattice with 800 sites, $\gamma =0.0$ and open-boundary conditions for one direction at a time, see Fig. 10 (refer to ’Methods’ section for details).

Physically, the term that distinguishes between Eqs. (1) and (9) restores the penetration depth of the surface state along the *z* direction with open-boundary conditions. Thereby, the projection of the Weyl points can be found in all planes. The collision between the Weyl points, as the control parameter $\gamma$ changes, occurs in the plane $k_{x}-k_{z}$ for $k_{y}=0$ (into the bulk), that contains simultaneously the red and blue spheres in Fig. 4.

To better understand the trajectory of the Weyl points in plane $k_{x}-k_{z}$ shown in Fig. 4b, we track in Fig. 5 the path followed by each of these points until the critical point at $\gamma =1$. These points reaarange their trajectories, such that they can collide along the $k_z$ direction.

**Figure 6:**
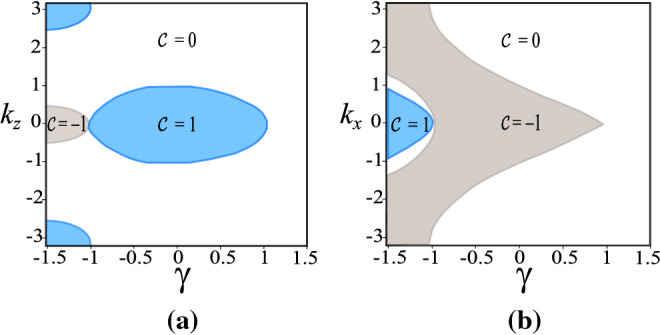
Phase diagrams and sector Chern number ${\mathscr {C}}$. (**a**) ${\mathscr {C}}(k_z)$ and (**b**) ${\mathscr {C}}(k_x)$ where the blue area stands for ${\mathscr {C}}=1$ and the gray one for ${\mathscr {C}}=-1$. The ${\mathscr {C}}(k_x)=0$ denotes a trivial topological phase^47,48^.

In order to characterize the topological phases and the topological phase transitions, we use the invariant sector Chern numbers^51,52^ ${\mathscr {C}}(k_x)$, ${\mathscr {C}}(k_y)$ and ${\mathscr {C}}(k_z)$. This invariant is given by ${\mathscr {C}}(k_{\mu })=\int _{BZ} dk_\rho dk_\lambda F_\mu$ with Berry curvature $F_\mu = \frac{1}{4 \pi } \epsilon _{\mu , \nu , \tau } h_{\mu } \partial _{\rho }h_{\nu } \partial _{\lambda }h_{\tau }/E_{+}^3$ (refer to ’Methods’ section for details). Here, the indexes $\rho$ and $\lambda$ are the orthogonal directions to the $\mu$, $\epsilon _{\mu , \nu , \tau }$ is the Levi-Civita antisymmetric tensor and BZ denotes the Brillouin Zone. The topological phases are distinguished by the Chern numbers, ${\mathscr {C}}=({\mathscr {C}}(k_x),{\mathscr {C}}(k_y),{\mathscr {C}}(k_z))$. In our case, depending on $\gamma$, $k_x$, $k_y$ and $k_z$ the sector Chern number can be equal to ${\mathscr {C}}(k_\mu )=0,-1,1$, see Fig. 6. Figure 6a,b show three different topological regions, the blue region possesses ${\mathscr {C}}(k_z)=1$ (non-trivial), the gray region ${\mathscr {C}}(k_z)=-1$ (non-trivial) and the white region has ${\mathscr {C}}(k_{z})=0$ (trivial).

**Figure 7:**
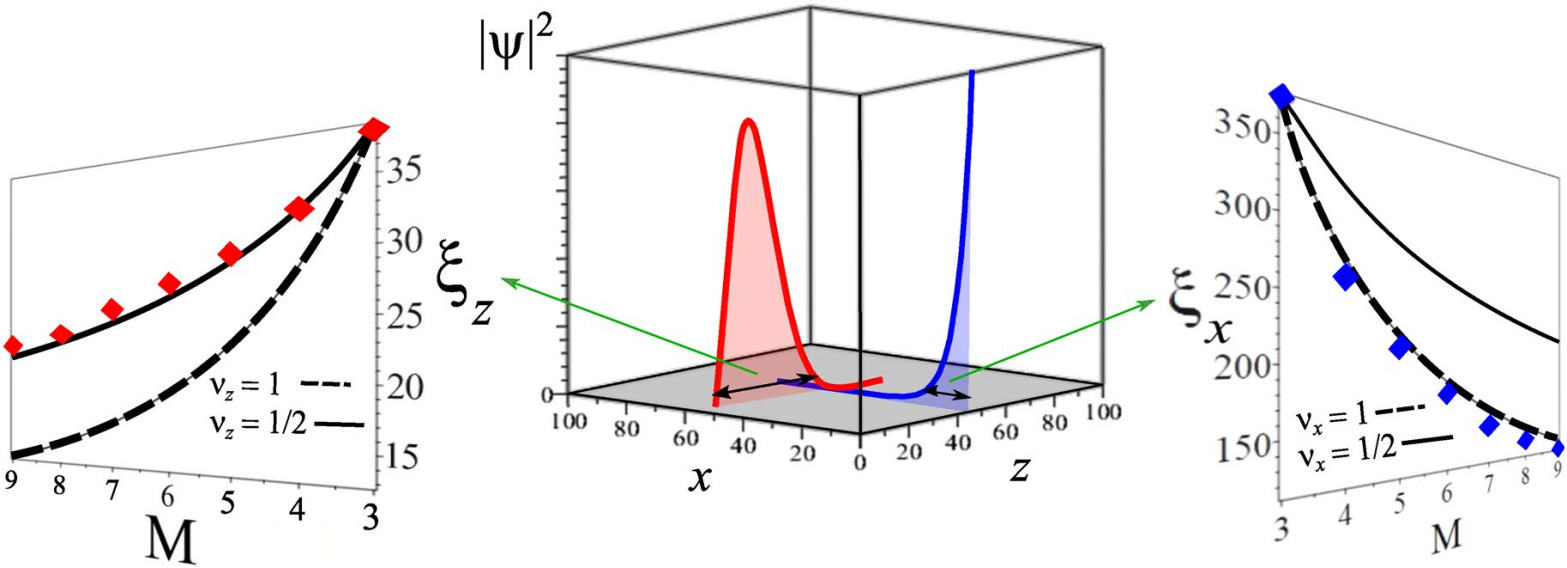
Probability density $|\psi |^{2}$ and penetration depth $\xi _{z}$ and $\xi _{x}$ as a function of $M\times 10^{-3}$ for the Hamiltonian Eq. (9). (middle figure) In cubic perspective, we show the density probability decay behavior for the cases with open-boundary conditions along *x* (light blue color) and *z* (light red color) directions, as indicated by the axis that represent the sites along each direction. (left figure) The results from open-boundary conditions along the *z* direction $\xi _{z}$ (red diamonds) are well described by a correlation length $\xi _z \propto M^{-\nu _z}$ with $\nu _{z}=\frac{1}{2}$, as shown by the black solid line. (right figure) The correlation length $\xi _x$ with open-boundary conditions along the *x* direction (blue diamonds) is in turn well described by $\nu _{x}=1$, see the black dashed line^47,48^.

Note that, in Fig. 6 the edges of the region blue in Fig. 6a and the region gray in Fig. 6b correspond to the coordinates of $k_{z}$ and $k_{x}$, respectively, of the Weyl points for each fixed $\gamma$. Accordingly, the projection of these coordinates on the surface delimits the Fermi arc extension, as represented in Fig. 4. Therefore, the topological regions in Fig. 6 are intimately related with the existence of the surface states. For instance, the surface states obtained for open-boundary conditions along *x* direction arise only for $k_{y}=0$ and for $k_{z}$ given by the blue region in Fig. 6a. This solution for the surface states corresponds on the Fermi arcs in Fig. 4 localized at $k_{y}=0$ where the plane $k_{x}-k_{z}$ shelters the Weyl points projection (yellow stripe). On the other hand, the surface states with open-boundary conditions only along *z* direction arise only for $k_{y}=0$ and for $k_{x}$ given by the gray region in Fig. 6b. Finally, we also consider open-boundary conditions along *y* direction to obtain the Fermi arcs numerically. These zero energy solutions are highlighted by the yellow stripe in Fig. 10c (refer to ’Methods’ section for details), and depicted in Fig. 4b along the plane $k_{x}-k_{z}$ (light blue plane).

In order to study the penetration depth $\xi$ of the zero energy Fermi arcs, we consider the region parameter around the critical point $\gamma =1$ for open-boundary conditions along the anisotropic *x* and *z* directions. In Fig. 7, we present the density probability $|\psi |^{2}$ obtained applying open-boundary conditions in both directions (middle figure). In cubic perspective, we highlight the density probability for *z* open-boundary conditions in red color and for *x* open-boundary conditions in blue color. The decay behavior is drastically different, which is reflected in the penetration depth quantities $\xi _{z}$ and $\xi _{x}$. In the left figure for *z* open, we can observe the penetration depth $\xi _{z}$ (red diamonds) as a function of the distance to the critical point *M*. The exponent $\nu _{z}=\frac{1}{2}$ gives an excellent fit with $\xi _{z}=\xi _{0}|M|^{-\frac{1}{2}}$ (black solid line). Otherwise, in the right figure, for *x* open, the decay of the penetration depth $\xi _{x}$ inside the bulk (blue diamonds) is very well fitted by $\xi _{x}=\xi _{0}|M|^{-1}$ (black dashed line), i.e., with $\nu _{x}=1$. So, we are able to identify the anisotropic critical exponent^41^ as $\theta =\nu _{x}/\nu _{z}=2$, where *x* and *z* are the current anisotropic directions.

## Universality class for Hopf insulators: beyond $z=2$ dynamic critical exponent

The Hopf topological insulator is a truly 3D topological insulator. It is an example of IT phases, which do not require any other symmetry protection beyond the U(1) charge conservation that is present in all kinds of TIs^22,23^. It is important to note that the Hopf insulator can only be realized in a two band Hamiltonian due to its non-trivial mapping from three-sphere to the two-sphere^22,42^. From the point of view of the Wannier representation, the two-band Hopf insulator can be recognized as a fragile topological system^53^ since, simply adding other bands while preserving gap and symmetries trivializes it. On the other hand, in Ref.^54^ the authors provide a Hopf multi-band generalization that is topological under the addition of the $\mathscr {C^{\prime }}$ generalized particle-hole symmetry, such that $J^{-1}HJ=-H^{*}$. We also verify the presence of the $\mathscr {C^{\prime }}$ for the two-band case, but it is absolutely inherent to the non-trivial mapping that characterizes the Hopf insulator in the first place. Note that, the Hopf insulator remains a no-symmetry protected topological insulator due to the dependence on mapping parameters p and q, as we discuss below. Remarkably, the $\mathscr {C^{\prime }}$ symmetry is claimed to protect the surface states in the multi-band generalization, while this is not the case in the two-band model. For instance, consider a general band Hamiltonian with *m* and *n*, filled and empty bands, respectively. In this case for *m* and *n* different from unit, the space Hamiltonian is mapping into its topologically equivalent Grassmannian manifold and consequently follows the homotopy group Grassmannian classification^43^. For 2 dimensions the homotopy group is $\pi ^2[G_{m,m+n}({\mathbb {C}})]={\mathbb {Z}}$ and classified by the Chern number. For 3D dimensions and when no additional discrete symmetries are required, the homotopy group is $\pi ^3[G_{m,m+n}({\mathbb {C}})]$ that corresponds only to the identity element. For this reason there is no winding number correspondence in 3D space dimensions^20,55^. However, for the special case of a two band Hamiltonian, i.e., $m=n=1$ the topologically equivalent space is ${\mathbb {S}}^2$. In this way, for the two band case we have an accidental winding number since the mapping comes from ${\mathbb {T}}^3$, a 3D Brillouin Zone torus, to the Grasmannian space parameter ${\mathbb {S}}^2$^20,22^. The non-trivial mapping construction has direct impact on the topological invariant called Hopf index $\chi$^42,56^. In Ref.^22^, the authors show that all Hopf insulators can be realized by the following Hamiltonian, ${\mathscr {H}}=h(k)\cdot \sigma$, where

10 $$\begin{aligned} h_{x}= & {} \mathfrak {Re}\left[ 2n_{\uparrow }^p {\overline{n}}_{\downarrow }^q\right] \end{aligned}$$

11 $$\begin{aligned} h_{y}= & {} \mathfrak {Im}\left[ 2n_{\uparrow }^p {\overline{n}}_{\downarrow }^q\right] \end{aligned}$$

12 $$\begin{aligned} h_{z}= & {} |n_{\uparrow }|^{2p}-|n_{\downarrow }|^{2q}, \end{aligned}$$

with

$$\begin{aligned} n_{\uparrow }= & {} \sin {k_{x}}+it\sin {k_{y}} \\ n_{\downarrow }= & {} \sin {k_{z}}+i(\cos {k_{x}}+\cos {k_{y}}+\cos {k_{z}}+h). \end{aligned}$$

Here, for each pair [*p*, *q*] we have a counterpart tight-binding Hamiltonian in real space which is also able to describe a Hopf insulator model if *p* and *q* are prime to each other. We have performed a study of the energy spectra for values of *p*, and *q* from $[p,q]=[1,1]$ to [8, 8]. For all cases, we identify the gap-closing points at critical parameter $h_{c}=-3,-1,1,3$ following the high-symmetry points of Eq. (40). The Hopf index is expected to be $\chi =0$ for $|h|>3$, $\chi =\pm pq$ for $1<|h|<3$ and $\chi =\pm 2 pq$ for $|h|<1$^22^. The non-zero Hopf indexes ensure the presence of gapless surface states in the Hopf insulator phase.

Accordingly, if the dispersion still depends on the critical exponents *z* and $\nu$, as well as the distance to the critical point $M=h-h_{c}$ we can write

13 $$\begin{aligned} E=\sqrt{M^{2\nu _{\bar{\alpha }} z_{\bar{\alpha }}}+\delta k_{\bar{\alpha }}^{2z_{\bar{\alpha }}}}, \end{aligned}$$

for each, $$\bar{\alpha }=x, y ,z$$.

**Figure 8:**
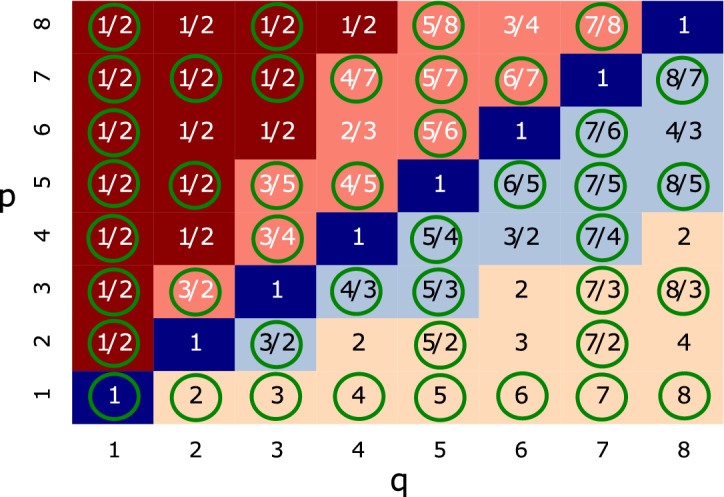
Heatmap of $\nu _{x}$ as a function of p and q values. Inserted values indicate $\nu _{x}$ for each pair [*p*, *q*], from 1 to 8. For $q< \frac{p}{2}$, $\nu _{x}=1/2$ while for $q\ge \frac{p}{2}$ we have $\nu _{x}=q/p$. For all values investigated, $\nu _{x}=\nu _{y}$ and $\nu _{z}$ is always equal to unit. The green circles indicate the values related to the topological Hopf insulator phase, i.e., *p* and *q* prime to each other^57^.

In order to study the gap-closing behavior as $M \rightarrow 0$, we put all $\mathbf {k}$ wavevectors at the corresponding high-symmetry points for a specific $h_{c}$ in Eq. (13). This yields $E=M^{\nu _{\bar{\alpha }} z_{\bar{\alpha }}}$, and we obtain, for all [p,q] values studied,

14 $$\begin{aligned} \nu _{\bar{\alpha }} z_{\bar{\alpha }}=2q. \end{aligned}$$

This is a critical exponent relation, independent of $h_{c}$, as expected. For instance, we get $\nu _{\bar{\alpha }} z_{\bar{\alpha }}=2$ for $[p,q]=[1,1],\,[4,1]$ and $\nu _{\bar{\alpha }} z_{\bar{\alpha }}=4$ for $[p,q]=[1,2],\,[3,2]$, as we can check in Eqs. (41) and (42) for $[p,q]=[1,1]$ and [1, 2], respectively. To evaluate each $z_{\bar{\alpha }}$, for example $z_{x}$, we put $h=h_{c}$, $\delta {k_{y}}=0$ and $\delta {k_{z}}=0$ to study the behavior only along *x* direction, which leads to $E\propto \delta k_{x}^{z_{x}}$. So, in general we expect $E\propto \delta k_{\bar{\alpha }}^{z_{\bar{\alpha }}}$ for each direction where $z_{\bar{\alpha }}$ is the dominant term around the critical point. We expand the energy around the critical values of *h*, for $[p,q]=[1,1]$ and [1, 2] (refer to ’Methods’ section for details). In each case we can check the dominant term for each direction and we get $z_{x}=2$ for both cases. If we know $z_{x}$ we are able to evaluate $\nu _{x}$ from Eq. (14). In this way we obtain $\nu _{x}=1$ and $\nu _{x}=2$ for $[p,q]=[1,1]$ and [1, 2], respectively. We perform the same steps for all [*p*, *q*] studied here and summarize the results in Fig. 8. The heatmap in Fig. 8 presents the $\nu _{x}$ for the corresponding [*p*, *q*]. Beyond that, we always find the symmetry $\nu _{x}=\nu _{y}$ and $\nu _{z}=1$. We also observe that the values of $\nu _{x}$ follow another critical exponent relation given by,

15 $$\begin{aligned} \nu _{x}= \left\{ \begin{array}{ll} 1/2, &{} \hbox {for}~{ q<p/2;} \\ q/p, &{} \hbox {for}~{ q\ge p/2.} \end{array} \right. \end{aligned}$$

For the present Hopf model, the anisotropic critical exponent^41^ can be written as $\theta =\nu _{x}/\nu _{z}=2$ for $q<p/2$ and $\theta =\nu _{x}/\nu _{z}=q/p$ for $q\ge p/2$, since $\nu _{z}=1$ in both cases. Once again, *x* and *z* are the current anisotropic directions.

**Figure 9:**
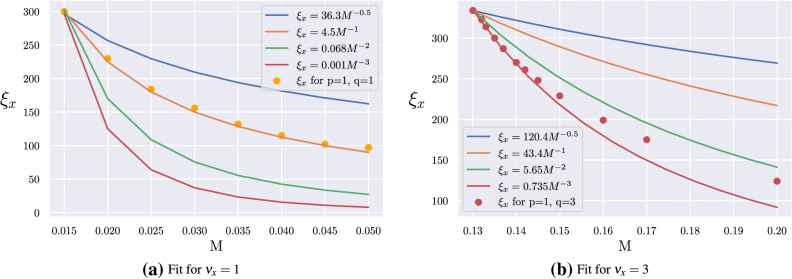
Penetration depth for the Hopf Hamiltonian. (**a**) For $[p,q]=[1,1]$ the best fit indicates $\nu _x=1$, and (**b**) For $[p,q]=[1,3]$ the best fit suggests $\nu _x=3$. These values of $\nu _x$ obtained numerically confirm the corresponding critical exponents given in Fig. 8.

We also obtain an anisotropic scaling relation for the Hopf insulator, $\theta =\nu _{x}/\nu _{z}=z_{z}/z_{x}$ (refer to ’Methods’ section for details), where the $x-y$ plane corresponds to ($\perp$) and the *z* direction to ($\parallel$). We derive an anisotropic quantum hyperscaling relation given by, $2-\alpha =\nu _{\perp }(z_{\perp }+d)-\nu _{\perp }(1-\theta )$ (Eq. (46)) (refer to ’Methods’ section for details). This relation connects the free energy critical exponent $\alpha$, to the dimension *d*, the correlation length critical exponent $\nu _{\perp }$, the dynamic exponent $z_{\perp }$ and the anisotropic critical exponent $\theta$.

Figure 8 gives very unusual values for the correlation length exponents. In order to verify these results, we obtain the penetration depths of the surface states. In Fig. 9a,b we give numerical evidence for $\nu _{x}=1$ and the more unusual value $\nu _{x}=3$, respectively. In order to illustrate the most suitable fit for these cases, we present in Fig. 9 some representative curves for different $\nu _{x}$ values using the expression $\xi _{x}=\xi _{0}M^{-\nu _{x}}$ (label of Fig. 9). For instance, we have $\nu _{x}=0.5,\,1,\,2$ and 3 for blue, orange, green and red solid lines. For the case $[p,q]=[1,1]$, the best fit in Fig. 9a is for $\nu _{x}=1$ (orange circles). In a similar way, for the case $[p,q]=[1,3]$, the best fit in Fig. 9b occurs for $\nu _{x}=3$ (red circles).

The Hopf surface states are zero-energy modes topologically protected and, as we see, present unusual values for the correlation length critical exponent $\nu$. We recall that this model corresponds to a tight-binding Hamiltonian, as long as *p* and *q* are integers prime to each other. The Hopf surface states also penetrate into the bulk, with a characteristic length that diverges at the critical point of the topological transition. In addition, it is expected that a larger Hopf index $\chi$ yields a larger number of surface states, but this correspondence remains unclear^42,58^.

## Discussion

The main purpose of the present work is to extend and characterize the critical behavior of topological materials that do not belong to the topological table of classification. We have studied two kinds of topological materials, the gapless Weyl semimetal and the no-symmetry protected, intrinsic topological material, the two-band Hopf insulator. The natural spacial anisotropy present in these models allow us to investigate an anisotropic scaling law from energy analysis and the penetration depth of the zero-energy surface states.

The first case we studied of an anisotropic band touching comes from the Weyl semimetal model, where the energy dispersions indicate $z_{y}=1$ and $z_{z}=2$ for the dynamic critical exponents along *y* and *z* directions, respectively. We proposed a modified Dirac equation that better translates this anisotropic aspect to calculate the wave-function of the surface states and its decay exponent. This investigation reveals a trivial, zero wave-function with open-boundary conditions along the *z* direction that contains the Weyl points. This precludes the evaluation a critical length exponent $\nu$ in this direction.

To overcome this difficulty, we propose a generalized Weyl semimetal model that includes the term $t_{a}\sin {k_{z}}\tau _{x}$ to the original model. This addition does not affect the anisotropic behavior, on the contrary, it promotes a rotation of the Weyl points around the $k_{y}$ axis, which enables the presence of Fermi arcs in all planes of the momentum space. The immediate consequence is the possibility to study the penetration depth with open-boundary conditions along all directions. From this, we verify $\nu _{x}=\nu _{y}=1$ and $\nu _{z}=1/2$, in agreement with previous results $z_{x}=z_{y}=1$ and $z_{z}=2$. We also can determine the anisotropic critical exponent $\theta =\nu _{x}/\nu _{z}=2$. In general, additional terms in the Weyl Hamiltonian ${\mathscr {H}}_{Weyl}(k)$ of Eq. (1) can change the values of the critical exponents, as in the case of different energy dispersion relations at the touching of the bands. However, as long as the anisotropic character of the Hamiltonian is preserved, the scaling law for the anisotropic critical exponent $\theta =\nu _{x} / \nu _{z}$ remains valid. The obtained non-zero sector Chern numbers evidence the non-trivial topological character of these systems. Furthermore, the boundary of region with a non-trivial Chern number in the phase diagram of Fig. 6 is associated with the extension of the Fermi arcs.

In order to go further, we investigate the Hopf insulator model. The non-trivial mapping leads to a tight-binding Hamiltonian for each pair of parameters [*p*, *q*], as long as *p* and *q* are integers prime to each other. From an analysis of the energy dispersion relations, we were able to determine the critical exponents $\nu _{\bar{\alpha }}$ and $z_{\bar{\alpha }}$ for each $\bar{\alpha }$ direction. The values of $\nu _{x}$ are presented in Fig. 8 and we discover they follow a pattern summarized in the scaling relation, $\nu _{\bar{\alpha }}z_{\bar{\alpha }}=2q$. Also we find that the $x-y$ plane is isotropic, such that $\nu _{x}=\nu _{y}=\nu _{\perp }=1/2$ for $q<p/2$ and $\nu _{x}=\nu _{y}=\nu _{\perp }=q/p$ for $q\ge p/2$, while in the *z*-direction we always have $\nu _{z}=\nu _{\parallel }=1$. The anisotropic critical exponent is obtained as $\theta =\nu _{x}/\nu _{z}=\nu _{\perp }/\nu _{\parallel }=z_{z}/z_{x}$ and leads to a quantum hyperscaling relation given by $2-\alpha =\nu _{\perp }(z_{\perp }+d)-\nu _{\perp }(1-\theta )$. In this hyperscaling relation, $\alpha$ is the exponent characterizing the singular behavior of the free energy ($f \propto |\delta |^{2- \alpha }$). Notice, that these results depend only on the Hamiltonian parameters *p* and *q* and the anisotropic directions. Indeed, this scaling law holds only for the two-band Hopf insulator, since any additional term may immediately disrupt the very restrict non-trivial mapping that characterizes this model.

Far away from the usual, we obtain exotic values for the critical exponents of the Hopf insulator. For instance, consider $p=1$ and $q=3$ where the heatmap of Fig. 8 predicts $\nu _{x}=3$. In fact, we can check this result obtaining the penetration depth and confirm that $\nu _{x}=3$ as shown in Fig. 9b. From the proposed scaling relation, we obtain $\nu _{x}z_{x}=3\cdot z_{x}=6$ that renders $z_{x}=2$. Accordingly, using this Hopf scaling law, together with the table of Fig. 8, it is possible to find $z_{\bar{\alpha }}=2,\,4,\,6,\,8,\,10$ and even higher values can arise if we extend the table for high values of *p* and *q*. In principle, we have not found an upper limit for the Hamiltonian parameters.

We can affirm, based on our results, that even the TPTs beyond the standard classification may support a connection with the theory of quantum critical phenomena. Furthermore, although surface states with non-zero-energy can exist, only the zero-energy states that are those obeying the bulk-boundary correspondence, allow to make the bridge between the TPT and critical phenomena. The properties of these states are unique and they can yield the set of critical exponents $\nu$ and *z* associated with the spacial and temporal correlations, respectively.

## Methods

### Low energy Weyl model

The expansion of the Weyl model close to the critical point of the topological transition yields,

16 $$\begin{aligned} H(k) = t_{x}k_{x}\tau _{x}+t_{y}k_{y}\tau _{y}+t_{z}\left[ \frac{k_{x}^{2}}{2}+\frac{k_{y}^{2}}{2}\right. + \left. \sin {k_{0}}(k_{z}-k_{0})-\frac{\cos {k_{0}}}{2}(k_{z}-k_{0})^{2}\right] \tau _{z}. \end{aligned}$$

From Eq. (16) the energy dispersion is given by,

17 $$\begin{aligned} E(k)=\pm \sqrt{t_{x}^{2}k_{x}^{2}+t_{y}^{2}k_{y}^{2}+t_{z}^{2}\left[ \frac{k_{x}^{2}}{2}+\frac{k_{y}^{2}}{2}+\sin {k_{0}}(k_{z}-k_{0})-\frac{\cos {k_{0}}}{2}(k_{z}-k_{0})^{2}\right] ^{2}}. \end{aligned}$$

### Topological surface state study for Weyl model

#### Surface state wave-function along *x* direction

We can introduce open-boundary conditions in *x* direction. In this case, it is interesting to treat the terms that depend on *x* (real space) as a problem in one-dimension, while the terms depending on $k_{y}$ and $k_{z}$ as a perturbation to the one-dimension problem. From Eq. (5), Since $k_{x}$ is the direction we apply open-boundary conditions, we isolate the Pauli matrices terms that include $k_{x}$

18 $$\begin{aligned} H(k)=\left\{ t_{x}k_{x}\tau _{x}+\left[ M-B\left( k_{x}^2+k_{y}^2+k_{z}^2\right) \right] \tau _{z}\right\} +t_{y}k_{y}\tau _{y}. \end{aligned}$$

The first term corresponds to the one-dimensional Hamiltonian $H_{k_{y},k_{z}}(x)=H_{1D}(x)$. The choice to include the quadratic terms on $H_{1D}(x)$ represents an *ansatz* so that the wave-function decays in the 1D open direction while oscillating in the other high dimensions considered. For this purpose, we propose the *ansatz* for the wave-function solution as

19 $$\begin{aligned} \psi (x,k_{y},k_{z})=\varphi _{k_{y},k_{z}}(x)\varphi (y,z). \end{aligned}$$

Then, applying this trial wave-function to Eq. (18), we have

20 $$\begin{aligned} \left[ t_{x}k_{x}\tau _{x}+\left( M-Bk_{x}^2-B{\bar{k}}^2\right) \tau _{z}\right] \varphi (x)=0 \end{aligned}$$

where $${\bar{k}}^2=k_{y}^2+k_{z}^2$$ and

21 $$\begin{aligned} t_{y}k_{y}\tau _{y}\psi (x,k_{y},k_{z})=\varepsilon \psi (x,k_{y},k_{z}). \end{aligned}$$

For Eq. (20) we take the zero-energy solutions, while in Eq. (21) we have a surface energy $\varepsilon$.

To solve Eq. (20), we multiply both sides by $\tau _{x}$ and put $k_{x}=-i\partial _{x}$ (open-boundary conditions for *x* direction). This leads to

22 $$\begin{aligned} t_{x}\partial _{x}\varphi (x)=-\left( M-B{\bar{k}}^2+B\partial _{x}^{2}\right) \tau _{y}\varphi (x). \end{aligned}$$

To avoid a trivial solution $\varphi (x)$, we assume that $\varphi (x)=\chi _{\eta }\phi (x)$ since $\tau _{y}\chi _{\eta }=\eta \chi _{\eta }$ with $\eta =\pm 1$, i.e., $\chi _{\eta }$ is an eigenvector of $\tau _{y}$. Then, Eq. (22) becomes

23 $$\begin{aligned} t_{x}\partial _{x}\phi (x)=-\eta \left( M-B{\bar{k}}^2+B\partial _{x}^{2}\right) \phi (x). \end{aligned}$$

The part of the wave-function that depends on *x* must possess a decay behavior, so $\phi (x)=e^{-\lambda x}$ where $\lambda >0$ ensures the decay. Following this, the equation for the characteristic length is given by

24 $$\begin{aligned} B\lambda ^2-\eta t_{x}\lambda + (M-B{\bar{k}}^2)=0 \end{aligned}$$

Then, the solutions for $\lambda$ are

25 $$\begin{aligned} \lambda =\frac{\eta t_{x}}{2B} \pm \frac{1}{2B}\sqrt{t_{x}^{2}-4B(M-B{\bar{k}}^2)} \end{aligned}$$

Since $\lambda >0$ and assuming that $t_{i}$ ($i=x,y,z$) are always positive, we have $\eta =sgn(B)$. Thus,

26 $$\begin{aligned} \lambda _{\pm }=\frac{t_{x}}{2|B|}\left[ 1 \pm \sqrt{1-\frac{4MB}{t_{x}^{2}}+\frac{4B^{2}{\bar{k}}^{2}}{t_{x}^{2}}}\right] . \end{aligned}$$

So, we take the general solution for $\phi (x)$ that satisfies the Dirichlet boundary conditions, i.e., $\phi (\pm \infty )=0$ and $\phi (0)=0$,

27 $$\begin{aligned} \phi (x)=e^{-\frac{x}{\xi _{+}}}-e^{-\frac{x}{\xi _{-}}} \end{aligned}$$

where $\lambda _{\pm }=\xi {\pm }^{-1}$ to recover the usual representation of the penetration depth $\xi _{\pm }$.

The eigenvector $\chi _{\eta }$ for $\eta =sgn(B)$ is given by

28 $$\begin{aligned} \chi _{\eta ,xo}=\frac{1}{\sqrt{2}}\left( \begin{array}{c} \eta \\ i \\ \end{array} \right) = \frac{1}{\sqrt{2}}\left( \begin{array}{c} sgn(B) \\ i \\ \end{array} \right) . \end{aligned}$$

The solution for $\varphi (y,z)$ can be found through Eq. (21). Since $\tau _{y}\chi _{\eta }=\eta \chi _{\eta }$, we have

29 $$\begin{aligned} t_{y}k_{y}\tau _{y}\psi (x,k_{y},k_{z})=\eta t_{y}k_{y}\psi (x,k_{y},k_{z}) \end{aligned}$$

that returns $\varepsilon =\pm sgn(B)t_{y}k_{y}$, the energy of surface state that reveals a current along the *y* direction with velocity $v_{y}=\frac{\partial \epsilon }{\partial k_{y}}=\pm sgn(B) t_{y}$. So, for the present model along the plane *yz* we have a current just along the *y* direction which is consistent with the Fermi arcs results. This also indicates that $\varphi (y,z)=\varphi (y)$, i.e., it does not depend on *z*. Thus, the wave-function with open-boundary conditions in *x* direction assumes the form

30 $$\begin{aligned} \psi (x,k_{y},k_{z})=\chi _{\eta ,xo}\left[ e^{-\frac{x}{\xi _{+}}}-e^{-\frac{x}{\xi _{-}}}\right] e^{i k_{y} y}. \end{aligned}$$

#### Surface state wave-function along *y* direction

In a similar way, for open-boundary conditions along *y* direction, we obtain the wave-function

31 $$\begin{aligned} \psi (y,k_{x},k_{z})=\chi _{\eta ,yo}\left[ e^{-\frac{y}{\xi _{+}}}-e^{-\frac{y}{\xi _{-}}}\right] e^{i k_{x} x}, \end{aligned}$$

where, $$\chi _{\eta ,yo}=\frac{1}{\sqrt{2}}\left( \begin{array}{c} -sgn(B) \\ 1 \\ \end{array} \right)$$ and

32 $$\begin{aligned} \lambda _{\pm }=\frac{t_{y}}{2|B|}\left[ -1 \pm \sqrt{1-\frac{4MB}{t_{y}^{2}}+\frac{4B^{2}{\bar{k}}^{2}}{t_{y}^{2}}}\right] , \end{aligned}$$

for $${\bar{k}}^{2}=k_{x}^{2}+k_{z}^{2}$$.

#### Surface state wave-function along *z* direction

Inspired by Eqs. (30) and (31), for open-boundary condition along the *z* direction, we propose

33 $$\begin{aligned} \psi (z,k_{x},k_{y})=\chi _{\eta ,zo}\left[ e^{-\frac{z}{\xi _{+}}}-e^{-\frac{z}{\xi _{-}}}\right] e^{i( k_{x} x+k_{y} y)}. \end{aligned}$$

Evaluating the Schrödinger equation for this wave-function at Dirichlet boundary conditions, i.e., $H(k)\psi (z,k_{y},k_{z})\mid _{z\pm \infty }=0$ where $k_{z}=-i\partial _{z}$, we have

34 $$\begin{aligned} \left( \begin{array}{ll} B(\lambda _{+}^{2}-\lambda _{-}^{2}) &{} 0 \\ 0 &{} -B(\lambda _{+}^{2}-\lambda _{-}^{2}) \\ \end{array} \right) \chi _{\eta ,zo}=\left( \begin{array}{c} 0 \\ 0 \\ \end{array} \right) . \end{aligned}$$

From the determinant of Eq. (34) the non-trivial solution returns $\lambda _{+}=\lambda _{-}$ or equivalently $\xi _{+}=\xi _{-}$. Following this, we can conclude that there is no decay wave-function with open-boundary conditions along *z* direction $\psi (z,k_{x},k_{y})=0$.

### Generalized Weyl semimetal: symmetries

The generalized Weyl model of Eq. (9) can be rewritten as

35 $$\begin{aligned} {\mathscr {H}}=h_{x}\sigma _{x}+h_{y}\sigma _{y}+h_{z}\sigma _{z} \end{aligned}$$

where $$h_{x}=t_{x}\sin {k_{x}}+t_{a}\sin {k_{z}}$$, $$h_{y}=t_{y}\sin {k_{y}}$$ and $$h_{z}=t_{z}\left( \gamma +2-\cos {k_{x}}-\cos {k_{y}}-\cos {k_{z}} \right)$$.

For the inversion symmetry, we have ${\mathscr {H}} \rightarrow {\mathscr {I}}{\mathscr {H}}(-k){\mathscr {I}}^{-1}$^39^ where ${\mathscr {I}}=\tau _{z}$ that yields

36 $$\begin{aligned} \tau _{z} \left( \begin{array}{cc} \scriptstyle {h_{z}} &{} \scriptstyle {-h_{x}+ih_{y}} \\ \scriptstyle {-h_{x}-ih_{y}} &{} \scriptstyle {-h_{z}} \\ \end{array} \right) \tau _{z}^{-1}= \left( \begin{array}{cc} \scriptstyle {h_{z}} &{} \scriptstyle {h_{x}-ih_{y}} \\ \scriptstyle {h_{x}+ih_{y}} &{} \scriptstyle {-h_{z}} \\ \end{array} \right) . \end{aligned}$$

So $\tau _{z}{\mathscr {H}}(-k)\tau _{z}^{-1}={\mathscr {H}}(k)$ and the Inversion symmetry is still preserved for the generalized Weyl model.

For the time-reversal symmetry, the symmetry operation reads ${\mathscr {H}} \rightarrow {\mathscr {T}}{\mathscr {H}}(-k){\mathscr {T}}^{-1}$. Since ${\mathscr {T}}=I{\hat{K}}$, where *I* is the identity matrix and ${\hat{K}}$ is the complex conjugate operator, we have

37 $$\begin{aligned} {\mathscr {T}} \left( \begin{array}{cc} \scriptstyle {h_{z}} &{} \scriptstyle {-h_{x}+ih_{y}} \\ \scriptstyle {-h_{x}-ih_{y}} &{} \scriptstyle {-h_{z}} \\ \end{array} \right) {\mathscr {T}}^{-1}= \left( \begin{array}{cc} \scriptstyle {h_{z}} &{} \scriptstyle {-h_{x}-ih_{y}} \\ \scriptstyle {-h_{x}+ih_{y}} &{} \scriptstyle {-h_{z}} \\ \end{array} \right) . \end{aligned}$$

Therefore, ${\mathscr {T}}{\mathscr {H}}(-k){\mathscr {T}}^{-1}\ne {\mathscr {H}}(k)$ and the time-reversal symmetry is still broken.

The stability of the two Weyl points requires one of these two symmetries to be broken. If this is not the case, and the material preserves simultaneously ${\mathscr {I}}$ and ${\mathscr {T}}$ we loose this stability and the two Weyl points with opposite topological charges merge leading to a zero total topological charge^39^.

### Topological surface state study for the generalized Weyl model

For the generalized Weyl model of Eq. (9) and following the same procedure adopted for open-boundary conditions along *x* direction, we have the presence of $t_{a}k_{z}\tau _{x}$ in Eq. (20). This yields $\lambda _{+} \ne \lambda _{-}$ in Eq. (34). For this reason, we can obtain non-trivial wave-functions for open-boundary conditions along any direction. From this, we can write $\lambda _{\pm }(k_{y},k_{z})$ in Eq. (38) and $\lambda _{\pm }(k_{x},k_{y})$ in Eq. (39), that stands for open-boundary conditions along *x* ad *z* directions, respectively. For these cases, the surface state energy remains $\varepsilon =\pm sgn(B)t_{y}k_{y}$, as for the Weyl semimetal model with open-boundary conditions along *x*.

38 $$\begin{aligned} \lambda _{\pm }(k_{y},k_{z})= & {} \frac{t_{x}}{2|B|}\left[ 1 \pm \sqrt{1-\frac{4MB}{t_{x}^{2}}+\frac{4B^{2}(k_{y}^{2}+k_{z}^{2})}{t_{x}^{2}}-\frac{4|B|t_{a}k_{z}}{t_{x}^{2}}}\right] \end{aligned}$$

39 $$\begin{aligned} \lambda _{\pm }(k_{x},k_{y})= & {} \frac{t_{a}}{2|B|}\left[ 1 \pm \sqrt{1-\frac{4MB}{t_{a}^{2}}+\frac{4B^{2}(k_{x}^{2}+k_{y}^{2})}{t_{a}^{2}}-\frac{4|B|t_{x}k_{x}}{t_{a}^{2}}}\right] \end{aligned}$$

**Figure 10:**
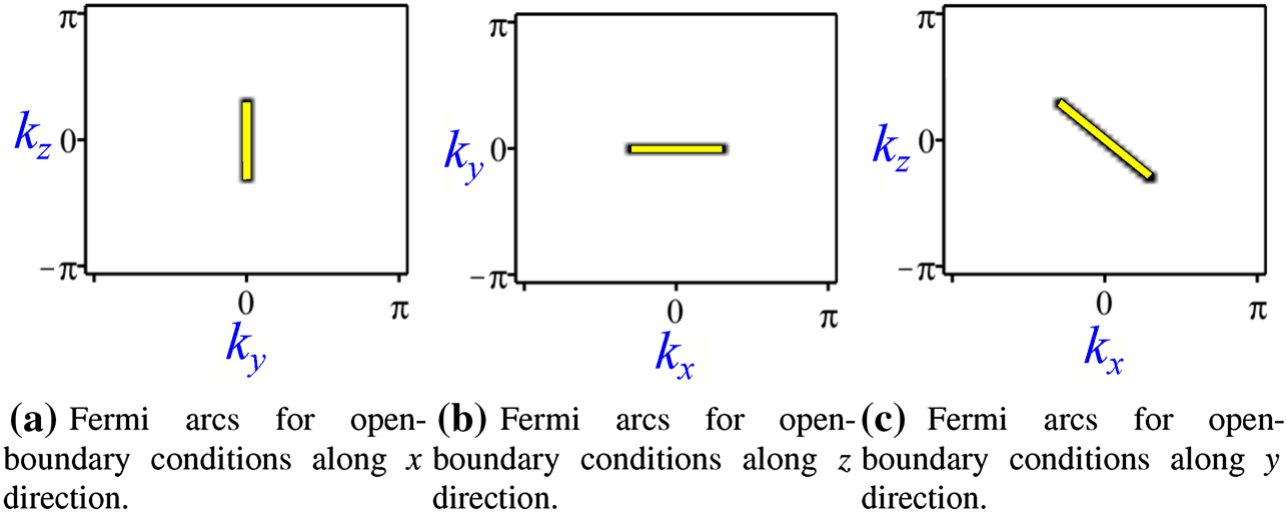
Fermi arcs obtained numerically for the generalized Weyl semimetal model^47^.

Accordingly, for open-boundary conditions along *y* direction, the $\lambda _{\pm }(k_{x},k_{z})$ preserve the same form obtained for the Weyl model under the same conditions. On the other hand, in this last case the surface state energy assumes the form $\varepsilon =\pm sgn(B)(t_{x}k_{x}+t_{a}k_{z})$. We also obtain the Fermi arcs numerically for the generalized Weyl model from the diagonalization of the Hamiltonian, Eq. (9), for a lattice with 800 sites, see Fig. 10. We report that the numerical results are in agreement with those obtained via the topological surface state study above. In fact, Fig. 10a,b present the zero-energy $\varepsilon =\pm sgn(B)t_{y}k_{y}=0$ for $k_{y}=0$ that describe the Fermi arc as expected. However, in Fig. 10c the zero-energy mode occurs for $\varepsilon =\pm sgn(B)(t_{x}k_{x}+t_{a}k_{z})=0$ that yields $k_{z}=-(t_{x}/t_{a})k_{x}$, where $-(t_{x}/t_{a})$ is the angular coefficient of the linear Fermi arc in the plane $k_{x}-k_{z}$ for $t_{x}=1$, $t_{a}=\sqrt{|1-\gamma |}=1$ and $\gamma =0$. It is important to emphasize, that the extension of the Fermi arcs still depends on the conditions that require that the correspondent $\lambda$ must be a real and positive quantity.

### Berry curvature components

The components of the Berry Curvature are

$$\begin{aligned} F_x= & {} \frac{[t_a(g+2-C_x)C_z - S_x S_z -t_a] C_y -t_a C_z}{2[(S_x+t_a S_z)^2+S_y^2+(g+2-C_x- C_y - C_z )^2]^{3/2}} \\ F_y= & {} -\frac{S_y(S_x C_z t_a-S_z C_x)}{2[(S_x+t_a S_z)^2+S_y^2+(g+2-C_x- C_y - C_z )^2]^{3/2}} \\ F_z= & {} \frac{((g-C_z+2)C_x-S_x S_z t_a-1)C_y-C_x}{2[(S_x+t_a S_z)^2+S_y^2+(g+2-C_x- C_y - C_z )^2]^{3/2}} \end{aligned}$$

where $$t_a=\sqrt{|1-\gamma |}$$, $$S_i=\sin k_i$$ and $$C_i=\cos k_i$$.

### Hopf insulator p and q analysis

The Hopf insulator model can admit the following critical points $h_{c}$ at the high-symmetry points:

40 $$\begin{aligned} h_{c}=-3\Rightarrow & {} (k_{x},k_{y},k_{z})=(0,0,0) \nonumber \\ h_{c}=-1\Rightarrow & {} (k_{x},k_{y},k_{z})=(\pi ,0,0),(0,\pi ,0),(0,0,\pi ) \nonumber \\ h_{c}=+1\Rightarrow & {} (k_{x},k_{y},k_{z})=(\pi ,\pi ,0),(\pi ,0,\pi ),(0,\pi ,\pi ) \nonumber \\ h_{c}=+3\Rightarrow & {} (k_{x},k_{y},k_{z})=(\pi ,\pi ,\pi ). \end{aligned}$$

Expanding the energy dispersion relation close to the critical values $h_{c}$, we obtain the exponents of each $\delta k_{\bar{\alpha }}=k-k_{\bar{\alpha } c}$ in all $\bar{\alpha }$ directions. From this, we are able to evaluate which order of $\delta k$ dominates close to the critical point for each $\bar{\alpha }$ direction in Eq. (13).

For instance, for a pair $[p,q]=[1,1]$ (for a general $h_{c}$) and $[p,q]=[1,2]$ (at $h_{c}=-3$) we get,

- $$p=1$$ ; $$q=1$$

41 $$\begin{aligned} E_{n}= & {} (h-h_{c})^2+\left[ 1\mp (h-h_{c}) \right] (\delta k_{x}^2+\delta k_{z}^2)\nonumber \\&+ \left[ t^2\mp (h-h_{c})\right] \delta k_{y}^2 \nonumber \\&+ \frac{1}{2}\left[ (\pm \delta k_{x}^2)(\pm \delta k_{y}^2)+(\pm \delta k_{x}^2)(\pm \delta k_{z}^2) +(\pm \delta k_{y}^2)(\pm \delta k_{z}^2) \right] \nonumber \\&+\frac{1}{4}\left[ \delta k_{x}^4+\delta k_{y}^4+\delta k_{z}^4 \right] . \end{aligned}$$

where, $+$ stands for $k_{\bar{\alpha } c}=0$ and − for $k_{\bar{\alpha } c}=\pi$.

- $$p=1$$ ; $$q=2$$

42 $$\begin{aligned} E_{n[0,0,0]}= & {} (h-h_{c})^4-[2(h-h_{c})^3-1]\delta k_{x}^2 \nonumber \\&-[2(h-h_{c})^3-t^2]\delta k_{y}^2-2(h-h_{c})^3(h-h_{c}-1)\delta k_{z}^2 \nonumber \\&+\frac{3}{2}(h-h_{c})^2\left[ \delta k_{x}^4+\delta k_{y}^4\right] \nonumber \\&+\frac{1}{2}[(h-h_{c})^2+2(h-h_{c}-1)^2]\delta k_{z}^4 \nonumber \\&-\frac{1}{3}(h-h_{c})\left( \delta k_{x}^6+\delta k_{y}^6\right) -\frac{1}{2}(h-h_{c}-1)\delta k_{z}^6 \nonumber \\&+\frac{1}{16}\left( \delta k_{x}^8+\delta k_{y}^8+\delta k_{z}^8\right) \end{aligned}$$

where $h_{c}=-3$. The energy equation for a general $h_{c}$ is more complicated to observe as $q>1$.

### Quantum hyperscaling in anisotropic systems

Consider a Hopf insulator, with two isotropic directions in k-space, say $k_x$ and $k_y$ and with one special direction, the $k_z$ axis. Let us consider all constant equal to 1. The dispersion relation close to gap closing point and taking into account only the dominant powers can be written as:

43 $$\begin{aligned} E(k)= |\delta |^{\nu _{\perp } z_{\perp }} + k_{\perp }^{z_{\perp }}+k_{\parallel }^{z_{\parallel }} \end{aligned}$$

where $\perp$ stands for the directions in the plane and $\parallel$ for the anisotropic direction. This equation can be rewritten as

$$\begin{aligned} E(k)=|\delta |^{\nu _{\perp } z_{\perp }}\left( 1 + \frac{k_{\perp }^{z_{\perp }}}{|\delta |^{\nu _{\perp } z_{\perp }}}+\frac{k_{\parallel }^{z_{\parallel }}}{|\delta |^{\nu _{\perp } z_{\perp }}} \right) , \end{aligned}$$

which in turn yields

$$\begin{aligned} E(k)=|\delta |^{\nu _{\perp } z_{\perp }}\left( 1 + \left( \frac{k_{\perp }}{|\delta |^{\nu _{\perp }}}\right) ^{^{z_{\perp }}}+ \left( \frac{k_{\parallel }}{|\delta |^{\frac{\nu _{\perp } z_{\perp }}{z_{\parallel }}}}\right) ^{z_{\parallel }} \right) . \end{aligned}$$

Defining,

44 $$\begin{aligned} \nu _{\parallel }= \frac{\nu _{\perp } z_{\perp }}{z_{\parallel }}, \end{aligned}$$

such that the anisotropy exponent is

$$\begin{aligned} \theta =\nu _{\parallel }/\nu _{\perp }=z_{\perp }/z_{\parallel }, \end{aligned}$$

we get

45 $$\begin{aligned} E(k)=|\delta |^{\nu _{\perp } z_{\perp }}F\left( k_{\perp }\xi _{\perp },k_{\parallel }\xi _{\parallel }\right) \end{aligned}$$

where F(x,y) is a scaling function. Notice that, $\xi _{\perp }=|\delta |^{-\nu _{\perp }}$ and $\xi _{\parallel }=|\delta |^{-\nu _{\parallel }}$. Also notice from Eq. (44) that $\nu _{\parallel } z_{\parallel }=\nu _{\perp } z_{\perp }$. The total ground state energy is given by,

$$\begin{aligned} E_{tot}=\sum _k E(k)=|\delta |^{\nu _{\perp } z_{\perp }}\int d\mathbf{k}_{\perp } dk_{\parallel } F\left( k_{\perp }\xi _{\perp },k_{\parallel }\xi _{\parallel }\right) . \end{aligned}$$

Proceeding we obtain,

$$\begin{aligned} E_{tot}=\sum _k E(k)=\frac{|\delta |^{\nu _{\perp } z_{\perp }}}{\xi _{\perp }^{d-1} \xi _{\parallel }} \int d(\mathbf{k}_{\perp }\xi _{\perp }) dk_{\parallel } \xi _{\parallel } F\left( k_{\perp }\xi _{\perp },k_{\parallel }\xi _{\parallel }\right) . \end{aligned}$$

Finally,

$$\begin{aligned} E_{tot}=\sum _k E(k)=|\delta |^{\nu _{\perp } z_{\perp }+(d-1)\nu _{\perp }+\nu _{\parallel }} \int dX dY F\left( X,Y \right) . \end{aligned}$$

The anisotropic quantum hyperscaling relation is given by,

$$\begin{aligned} 2-\alpha =\nu _{\perp } z_{\perp }+(d-1)\nu _{\perp }+\nu _{\parallel } \end{aligned}$$

or

$$\begin{aligned} 2-\alpha =\nu _{\perp }( z_{\perp }+d) -\nu _{\perp }+\nu _{\parallel } \end{aligned}$$

Finally, using Eq. (44) we get

46 $$\begin{aligned} 2-\alpha =\nu _{\perp }( z_{\perp }+d) -\nu _{\perp }(1-\theta ) \end{aligned}$$

The exponent $\alpha$ gives the singular behavior of the free energy at the topological transition ($f \propto |\delta |^{2 - \alpha }$).
